# Mother-infant bonding is not associated with feeding type: a community study sample

**DOI:** 10.1186/s12884-019-2264-0

**Published:** 2019-04-11

**Authors:** Ilana S. Hairston, Jonathan E. Handelzalts, Tamar Lehman-Inbar, Michal Kovo

**Affiliations:** 1 Department of Psychology, Academic College of Tel-Hai, 1220800 Qiryat Shemona, Israel; 2 Psychiatry Department, University of Michigan, Ann Arbor, 48109 USA; 3 School of Behavioral Science, Academic College of Tel Aviv, Jaffa, Israel; 4 Obstetrics & Gynecology, Edith Wolfson Medical Center, Ha-Lochamim 62, Holon, Israel; 5 Affiliated with Sackler School of Medicine, Tel Aviv University, Tel Aviv, Israel

**Keywords:** Nursing, Bayesian statistics, Postpartum depression, Sleep disturbances

## Abstract

**Background:**

Bonding refers to emotions and cognitions towards one’s infant. Breastfeeding is believed to facilitate bonding, yet only a handful of studies have empirically tested this assertion. This study aimed to confirm whether a positive association between breastfeeding and bonding exists and whether breastfeeding may be protective against the negative consequences of mood and sleep disturbances on bonding.

**Method:**

A cross-sectional survey was administered to a convenience sample of Israeli mothers of infants ages 1–9 months. The main outcome measures were breastfeeding history, bonding (Postpartum Bonding Questionnaire, PBQ), mood (Edinburgh Postnatal Depression Scale, EPDS) and sleep (Pittsburgh Sleep Quality Index, PSQI).

**Results:**

Two hundred seventy-one mothers (21–46 years) completed the survey. 65.7% reported current breastfeeding, 22.1% past breastfeeding, 12.2% never nursed. The PBQ correlated with both the EPDS and PSQI. Breastfeeding was associated with greater daytime fatigue, but not with any other sleep problem, and was not associated with bonding. This negative result was confirmed with Bayesian analysis demonstrating that the probability for the null hypothesis was 4.5 times greater than the hypothesized effect. Further, hierarchical regression revealed a positive relationship between bonding, daytime fatigue and depression symptoms only among women who were currently breastfeeding.

**Conclusions:**

These findings suggest that among healthy mothers, breastfeeding may not be a central factor in mother-infant bonding, nor is it protective against the negative impact of mood symptoms and bonding difficulties. Theoretical and methodological bases of these findings are discussed.

**Electronic supplementary material:**

The online version of this article (10.1186/s12884-019-2264-0) contains supplementary material, which is available to authorized users.

## Background

The quality of the relationship between parents and their infant children plays a central role in psychological development. Mother-infant bonding is one aspect of this relationship, referring to the process in which a mother forms an affectionate attachment to her infant [1]. Originally conceptualized to occur within a “critical period” hours after birth, mediated by physical contact [2], bonding is now construed as ‘*an affective state’* of the parent*,* that emerges during pregnancy or immediately after birth, and continues to develop over the first months of the infant’s life, and can be assessed using maternal self-report instruments [3].

In line with Klaus and Kennell’s original bonding theory that emphasized physical proximity and skin-to-skin contact as necessary for maternal bonding [2], both lay and academic literature tend to favor breastfeeding as a vehicle for promoting maternal bonding and care [4, 5]. Indeed, breastfeeding naturally supplies the opportunity for skin-to-skin contact, and thus - in theory – should encourage affiliative emotions. And while the conceptualization of bonding has since evolved [3, 6], the importance of breastfeeding to bonding remains a stalwart belief in popular culture. Consistently, one of the most common reasons given by women for wanting to breastfeed is the opportunity to bond with their children [7–9] a belief also shared by health professionals [10, 11]. Further, the benefits of breastfeeding are actively promoted by public health organizations not merely as the healthiest nutritional choice, but also to “… *promote [s] the emotional relationship, or bonding, between mother and infant*.” [12]

Arguably, the notion that a link exists between maternal bonding and breastfeeding originates in cultural norms [13–15]. Although human mother’s milk has been the primary form of infant nutrition for thousands of years, in the absence of appropriate alternatives, wet nursing (AKA, adoptive breastfeeding), whether paid or via communal sharing of maternal responsibilities, was very common well into the eighteenth century [16]. Reduction in childbirth and infant mortality, and changes in social constructs of family and motherhood in the 19th and 20th centuries increased the likelihood that mothers and infants survived, and that mothers would breastfeed their babies, at least in the first few weeks. Nevertheless, while mother’s milk was deemed nutritionally superior, formula feeding was considered medically and socially acceptable during the first half of the twentieth century. Only in the later decades of the twentieth century, with the surgence of breastfeeding advocacy, which recommends exclusive breastfeeding for the first 6 months of life and beyond (e.g., ‘Breast is Best’) [14, 16], has breastfeeding also been linked with maternal affiliative bond to her child (e.g., [13, 17]).

However, only a handful of studies have directly tested the existence of a positive association between breastfeeding and bonding in humans with inconsistent results [4, 18]. In a recent longitudinal study, Nishioka, Haruna, Ota, Matsuzaki, Murayama, et al., (2011) [19] found that mothers who exclusively or nearly exclusively formula-fed their infants had a smaller increase in bonding feelings from one to 5 months postpartum compared with women who breastfed. In contrast, Else-Quest, Hyde & Clark (2003) [20] reported that the association between breastfeeding and bonding was weak at 4 months, and nonexistent at 12 months of age. Cernadas, Noceda, Barrera, Martinez, & Garsd (2003) [21] assessed bonding a few days after birth and prospectively correlated the measure with the duration of exclusive breastfeeding, up to 6 months. They found that early bonding predicted breastfeeding duration (rather than vise-versa). Finally, Martone and Nash (1988) [22], compared maternal emotional behaviors towards 2 day-old newborns during either bottle- or breast- feeding, and found no significant group differences. Thus, it remains unclear if breastfeeding considerably contributes to bonding among healthy mothers.

Difficulties with bonding have been reliably linked with depressed mood symptoms [23], an association partially explained by disrupted maternal sleep [24, 25]. The link between peripartum depressive symptoms and breastfeeding is bidirectional but suggests a negative relationship. Depression has been shown to increase the risk for early cessation of breastfeeding, while exclusive breastfeeding was associated with a more rapid decline in postpartum depression symptoms [26]. Further, women with high levels of depression during pregnancy, and who stopped breastfeeding early, were at additional risk for postpartum depression [27]. With respect to sleep, only a handful of studies have investigated the relationship with breastfeeding, with some reporting more sleep disruption in breastfed infants (e.g., [28]), while others reported positive [29] or no substantial impact [30] in comparison to bottle feeding. Thus, it is feasible that breastfeeding may be protective against the negative effects of mood and sleep problems on bonding by increasing the nurturing contact between mother an infant.

Hence, despite its theoretical, social and practical significance, the link between bonding and breastfeeding remains understudied. Therefore, this study aimed to directly assess the association between breastfeeding and bonding. As parent-infant bonding evolves over time, the current study cross-sectionally measured breastfeeding and bonding over a range of ages, from 1 to 9 months. To assess bonding, the Postpartum Bonding Questionnaire (PBQ) [31, 32] was used. The PBQ is a widely used, reliable and easy to administer instrument, which has been validated in several languages, including in Europe and the Middle East (e.g., [33–36]). While the instrument was developed to identify problems in the mother-infant relationship during the postpartum period, and higher scores reflect more bonding difficulties, it has been found to correlate with similar instruments that focus on positive aspects of bonding [37, 38].

The first hypothesis was that there will be a negative correlation between breastfeeding and bonding difficulties, as measured by the PBQ. Second, that this relationship would be age-dependent such that breastfeeding would have a greater impact on bonding during the first weeks of life, with a diminishing role among mothers of older infants, when other meaningful interactions come into play. Finally, as bonding is strongly linked with maternal mood and sleep difficulties, we further hypothesized that breastfeeding would be protective against the ill-effects of mood and sleep disturbances on mother-infant bonding.

## Methods

### Participants

Participants were recruited as part of a larger longitudinal study which aims to assess factors involved in the development of bonding over the first year of life. Online questionnaires were completed by mothers of infants 1–9 months of age (M = 4.2, SD = 2.3). Eligibility was restricted to healthy, full-term, infants. Inclusion criteria were the willingness to complete the questionnaires in full, exclusion criteria were premature birth (before week 36), and chronic illness of the infant.

### Instruments

#### Demographic questionnaire

Nineteen items provided information on socio-demographic details of women’s age, education, employment, marital status, income, number of children and infant’s age and gender.

#### Breastfeeding

Questions regarding breastfeeding included a question about the status of breastfeeding. There were three response options: exclusive = breastmilk only; partial = breastmilk and other foodstuffs, e.g., formula or solids; not breastfeeding. Women who responded that they are not currently breastfeeding were asked if they breastfed in the past, and if positive for how long. Breastfeeding duration was calculated according to infant’s age, hence infant’s age co-varied with breastfeeding duration and was held constant in statistical analyses.

#### Hebrew version of the postpartum bonding questionnaire (PBQ)

Mother-infant bonding was measured using the [31, 32], a reliable screen for mother–infant relationship disorders. The original questionnaire consisted of 25 items pertaining to the mother’s feelings and attitudes towards her infant. The questionnaire yields four subscales – a general factor, rejection, and pathological anger, anxiety about the infant, and incipient abuse. For ethical reasons, the ‘incipient abuse’ items were not included so that 23 items were included. Respondents rate agreement with statements on a 6-point Likert scale ranging from “0”-always to “5”-never. Thus, the lowest possible score is 0, the highest possible score for the total PBQ is 115, 60 for the general factor, 35 for rejection and pathological anger, and 20 for anxiety about the infant. Cutoff points for bonding disorders for the full scale are > 25, for the general factor subscale > 11, for rejection and anger subscale > 16, and for anxiety about care > 12 [32] Items were translated and back- translated in accordance with Brislin’s guidelines [24]. The internal consistency coefficient in this sample was (α = .914). As the PBQ is largely designed to assess bonding disorders, and the sample was a non-clinical sample, it remains possible that breastfeeding enhanced positive emotions and cognitions towards the infant but had little effect on negative ones. Thus, we created a scale using only positive items (e.g., “I feel close to my baby”; “I love my baby very much”; “I feel confident when changing my baby”), which yielded a fair internal consistency (α = 0.739).

*Hebrew version of The Edinburgh Postnatal Depression Scale* (EPDS, [39]) was used to assess mood symptoms in mothers. The EPDS is a 10-item instrument, specifically designed to address depressive symptoms in the postnatal period. The measure has been validated in childbearing women and has demonstrated high internal consistency and validity for detecting major depression in the perinatal period. A cutoff score to screen for major depression in postpartum women has been consistently found to be 13 or more [40]. Internal consistency in this sample was α = .851.

*Hebrew version of the Pittsburgh Sleep Quality Index* (PSQI, [41]) was used to assess mothers’ sleep. The PSQI is a self-rated instrument that evaluates sleep quality and disturbances over the past month. It has 19 individual items from which seven component scores, weighted equally on a 0–3 scale. The components relate to typical sleep/wake complaints, including: subjective sleep quality, sleep latency, sleep duration (or total sleep time, TST), habitual sleep efficiency, sleep disturbances, use of sleeping medications, and daytime dysfunction. The seven component scores are then summed to yield a global PSQI score, which has a range of 0–21, where higher scores indicate worse sleep quality. A cutoff score of 5 has been recommended, with scores > 5 indicating subjective insomnia [42]. The Internal consistency in this sample was α = 0.583. In addition to the PSQI, respondents were asked how much time they are awake at night on average, as a measure of wake after sleep onset (WASO).

### Procedure

The protocol and consent forms for the study was approved by the Helsinki committee of Edith Wolfson Medical Center and the Institutional Review Board of the Academic College of Tel Aviv – Yafo. The Internet-based survey was targeted at mothers of infants ranging from 1- to 9 months. Women were recruited either soon after birth at the maternity ward at Edith Wolfson Medical Center, or via internet ads published on parenting forums, relevant Facebook groups, and the snowball method. Informed consent was obtained online in the following manner: The first screen provided respondents with information regarding the aims and risks of the study, inclusion and exclusion criteria, and contact information of the authors. Upon reviewing this information, respondents were required to agree to participate before proceeding to the full survey. Agreement to participate and exclusionary questions were the only required responses in the survey. In return for completing the questionnaires in full, participants were provided a coupon of the equivalent value of $10 in Israeli shekels. All identifying details were omitted from the database used for analyses. Questionnaires and data output were generated using Qualtrics© 2015 (Qualtrics, Provo, UT, USA. http://www.qualtrics.com).

### Statistical analyses

Statistics tables and graphics were generated in SPSS V23. As multiple variables were included in the statistical models for hypothesis testing, chi-square distribution of Mahalanobis distance estimates, calculated using EPDS, total PBQ scores, and sleep symptoms, was used to remove outliers that exceeded the probability of 99.9%. Hypothesis testing was done using bootstrapped multivariate ANOVAs and linear regressions in SPSS. First-order correlation analyses, controlling for infant age, were used to determine the association between variables of interest (i.e., breastfeeding status, breastfeeding duration, PBQ and its subscales, EPDS and mother’s sleep variables). Stratified bootstrapping was used in ANOVA and regression analyses to adjust for the different Ns in the breastfeeding groups. JASP V0.9 was used for Bayesian testing to quantify evidence for the null (H0) and alternative (H1) hypotheses. The Bayesian approach to hypothesis testing considers the likelihood of the data under each hypothesis, allowing inferences regarding the distribution of the actual data. The statistic for comparing the probability of a set of observed data under two models is termed the Bayes Factor (BF). The nomenclature used is BF10, representing the odds for H1, or 1/BF10 (BF01), representing the odds for the null hypothesis. BF10 < 0.33 provides strong or ‘substantial’ evidence for the null hypothesis, BF10 > 3 provides strong evidence for the alternative (H1) hypothesis, between 0.33 and 3 provides only anecdotal support either way [43]. This analytical tool allows to infer the validity of the null hypothesis above and beyond the uncertainty of a non-significant value (i.e., insufficient evidence to reject the null hypothesis). Finally, moderation analyses were done using multiple hierarchical regressions.

## Results

Of 585 entries, 272 women completed the questionnaires in full, after removal of outliers 271 participants were included in the analysis. The sample was largely middle class and well educated (Table 1). Mean age of participants was 31.9 ± 4.2 (range: 21–46), and mean infant age was 4.3 ± 2.3 months (range: 1–9). Fifty-three percent of infants were female, 52% were the only child, 30% had one sibling, and the remainder had two or more siblings.

**Table 1:** Sample Characteristics

Demographic variables (*N* = 271)	statistic
Education %	
Less than 8 years	0.4%
8–12 years	8.5%
12–16 years	48.7%
16+	42.1%
With partner / married	95.9%
Employment	
Full-time employment	59.4%
Partial employment & Student	14.4%
Full-time student	7%
Neither	18%
Household income	
Significantly below median	9.2%
Below median	17.3%
Around median	34.3%
Above median	25.1%
Significantly above median	8.9%
Declined to answer	3.3%

Median monthly household income was equivalent to approximately 3715 USD at the time of data collection

Breastfeeding data are reported in Table 2. Eighty-seven and a half percent (87.5%) reported breastfeeding exclusively or partially. Respondents were grouped into currently breastfeeding (exclusive or partial, *N* = 178), past (*N* = 60), and never nursed (*N* = 33). Distribution of breastfeeding across infant ages is presented in the (Additional file 1: Figure S1). Postpartum Bonding Questionnaire (PBQ) scores were summed for the three subscales and the total PBQ scale. PBQ scores above 25 indicate some bonding disorder, with scores above 39 indicating severe bonding disorder [43]. In this sample, 91.9% were within normal range, while 1.8% (5 women) met criteria for severe bonding disorder. The Edinburgh Postnatal Depression Scale (EPDS) scores ranged from 0 to 25, with 7.7% of the respondent above the cutoff for clinical depression [40]. More than 64 % (64.2%) of participants scored above the cutoff of 5 for the Pittsburgh Sleep Quality Index (PSQI, [42]), although the sleep disturbance was minor. Respondents reported a short sleep period of about 6 h. on average, and frequent nighttime awakenings, with 57.9% reporting at least one nighttime awakening, and 20% two or more. On average, respondents reported being awake 101.2 min (0–240 min).

**Table 2:** Dependent variable in the study. Values represent means and standard deviations (in brackets)

	Exclusive	Partial	Past	Never	Total
	*N* = 129 (47.6%)	*N* = 49 (18.1%)	*N* = 60 (22.5%)	*N* = 33 (12.2%)	*N* = 271
PBQ total	10.5 (9.2)	13.4 (12.9)	10.9 (11.1)	12.6 (13.6)	11.4 (10.9)
general factor	6.4 (5.1)	7.4 (6.7)	6.4 (5.9)	6.9 (7.6)	6.5 (5.7)
rejection and anger	6.1 (2.2)	6.7 (2.5)	6.2 (2.9)	7.2 (3.1)	6.3 (2.5)
anxiety about the infant	2.3 (2.1)	3.7 (3.1)	2.7 (2.6)	3.1 (2.8)	2.7 (2.4)
EPDS	5.6 (4.1)	6.1 (5.2)	5.5 (4.6)	5.1 (4.2)	5.6 (4.4)
PSQI	7.0 (3.3)	6.8 (3.3)	6.9 (3.4)	6.8 (3.5)	6.9 (3.2)
Total sleep time (min)	367.2 (82.4)	374.1 (101.3)	364.8 (76.2)	368.6 (109.5)	367.9 (88.1)
Sleep onset latency (min)	19.3 (21.5)	18.2 (19.0)	26.8 (23.9)	22.7 (20.9)	21.2 (21.7)
Comp. #7 (range 0–3)	1.9 (1.4)	2.0 (1.5)	1.6 (1.3)	1.2 (1.2)	1.1 (0.8)
Comp. #1 (range 0–3)	1.3 (0.7)	1.3 (0.6)	1.4 (0.8)	1.4 (0.9)	1.4 (0.7)
WASO (min)	109.5 (59.4)	94.4 (61.4)	95.1 (69.1)	90.0 (70.7)	101.2 (63.5)

*PBQ* postpartum bonding questionnaire, *EPDS* Edinburgh Postnatal Depression Scale, *PSQI* Pittsburgh Sleep Quality Index, *Comp. #7* Daytime dysfunction items of the PSQI, *Comp. #* Overall sleep quality item of the PSQI, *WASO* wake after sleep onset

Table 3 depicts first-order correlation analyses, controlling for infant age, between demographic variables, breastfeeding, depression, bonding and sleep symptoms, using first-order correlation analysis controlling for infant age. As can be seen, older mothers reported higher income and more children. A higher income was also associated with better sleep (lower PSQI score and more TST). There were positive correlations among bonding difficulty scales (PBQ), sleep difficulties scales (PSQI), and depression (EPDS). Breastfeeding was associated with higher scores on the daytime fatigue/dysfunction component (component 7), but not with any of the other components of the PSQI, nighttime awakening or the EPDS. Refuting our first hypothesis, that breastfeeding would correlate with bonding, no relationship was observed between breastfeeding and the PBQ scales.

**Table 3:** First-order correlations controlling for infant age

		1	2	3	4	5	6	7	8	9	10	11
1. mother’s age	*R*	1										
	*p*	.										
2. Income	*R*	**0.346**	1									
	*p*	**<.001**	.									
3. First child	*R*	**−0.356**	−0.088	1								
	*p*	**<.001**	.159	.								
4. Breastfeeding groups	*R*	0.049	0.076	0.051	1							
	*p*	.425	.223	.404	.							
5. Breastfeeding duration	*R*	0.050	0.067	0.036	0.763	1						
	*p*	.419	.287	.559	<.001	.						
6. PBQ total	*R*	−0.017	−0.092	0.084	−0.027	− 0.024	1					
	*p*	.777	.140	.168	.656	.698	.					
7. PBQ general factor	*R*	−0.016	−0.089	0.079	0.002	−0.008	0.964	1				
	*p*	.801	.156	.193	.972	.895	<.001	.				
8. PBQ rejection and anger	*R*	0.020	−0.070	0.004	−0.085	−0.049	0.828	0.770	1			
	*p*	.741	.261	.954	.162	.423	<.001	<.001	.			
9. PBQ anxiety about child	*R*	−0.041	−0.049	**0.144**	−0.047	− 0.018	0.819	0.705	0.595	1		
	*p*	.512	.436	**.019**	.446	.763	<.001	<.001	<.001	.		
10. EPDS	*R*	0.049	−0.050	0.002	0.064	0.063	**0.531**	**0.513**	**0.423**	**0.476**	1	
	*p*	.427	.427	.969	.295	.305	**<.001**	**<.001**	**<.001**	**<.001**	.	
11. WASO	*R*	−0.047	−0.077	0.025	0.100	0.104	**0.172**	**0.200**	0.073	**0.154**	**0.298**	1
	*p*	.444	.220	.387	.106	.093	**.005**	**.001**	.235	**.013**	**<.001**	.
12. PSQI Total	*R*	0.030	**−0.137**	0.030	0.004	0.04	**0.173**	**0.202**	0.118	**0.136**	**0.453**	**0.476**
	*p*	.629	**.028**	.626	.944	.513	**.004**	**.001**	.052	**.026**	**<.001**	**<.001**
13. PSQI TST	*R*	−0.109	**0.132**	0.034	0.018	−0.062	− 0.057	−0.093	0.010	0.010	**−0.167**	**−0.369**
	*p*	.079	**.037**	.586	.773	.313	.352	.130	.876	.997	**.006**	**<.001**
14. PSQI SOL	*R*	−0.039	−0.051	0.053	−0.098	− 0.094	−0.009	− 0.008	−0.018	0.075	**0.185**	**0.221**
	*p*	.529	.425	.388	.114	.128	.886	.894	.767	.225	**.003**	**.001**
15. PSQI #7	*R*	0.062	−0.009	0.014	**0.181**	**0.134**	**0.288**	**0.292**	**0.221**	**0.205**	**0.413**	**0.188**
	*p*	.316	.887	.813	**.003**	**.028**	**<.001**	**<.001**	**<.001**	**.001**	**<.001**	**.002**
16. PSQI #1	*R*	0.020	−0.080	0.018	−0.038	−0.015	**0.161**	**0.178**	**0.159**	**0.14**	**0.366**	**0.427**
	*p*	.74	.201	.77	.538	.808	**.008**	**.003**	**.009**	**.022**	**<.001**	**<.001**

In bold are significant correlations; *PBQ* Postpartum Bonding Questionnaire, *EPDS* Edinburgh Postnatal Depression Scale, *WASO* Wake After Sleep Onset, *PSQI* Pittsburgh Sleep Quality Index – total score, *PSQI #7* daytime dysfunction component, *PSQI #1* overall sleep quality component, *TST* Total Sleep Time, *SOL* Sleep Onset Latency. Correlations among PSQI components were omitted

To test the likelihood of not rejecting the null hypothesis, a Bayes Factor (BF) linear regression was conducted with breastfeeding groups as the independent variable, infant age as a nuisance variable, and the total PBQ score as the dependent variable. It was found that the likelihood of the null hypothesis given the data was 4.5 times greater than for the alternative hypothesis (Table 4), considered within the range of *strong evidence* for the null hypothesis [44].

**Table 4:** Bayesian Linear Regression

Model Comparison: Total PBQ Score					
Models	P(M)	P(M|data)	BF _M_	BF _10_	error %
Null model (incl. Infant age)	0.500	0.819	4.518	1.000	
Breastfeeding	0.500	0.181	0.221	0.221	0.010

Breastfeeding variable with three levels (current | past | never). *P(M)* prior model probabilities, *P(M|data)* the updated probabilities after accounting for the data, *BF*_*M*_ Bayes Factor of the model, reflects the degree to which the data have changed the prior odds, *BF*_*10*_ odds for H1 (1/BF10 odds for null hypothesis). Model includes infant age

**Fig. 1:**
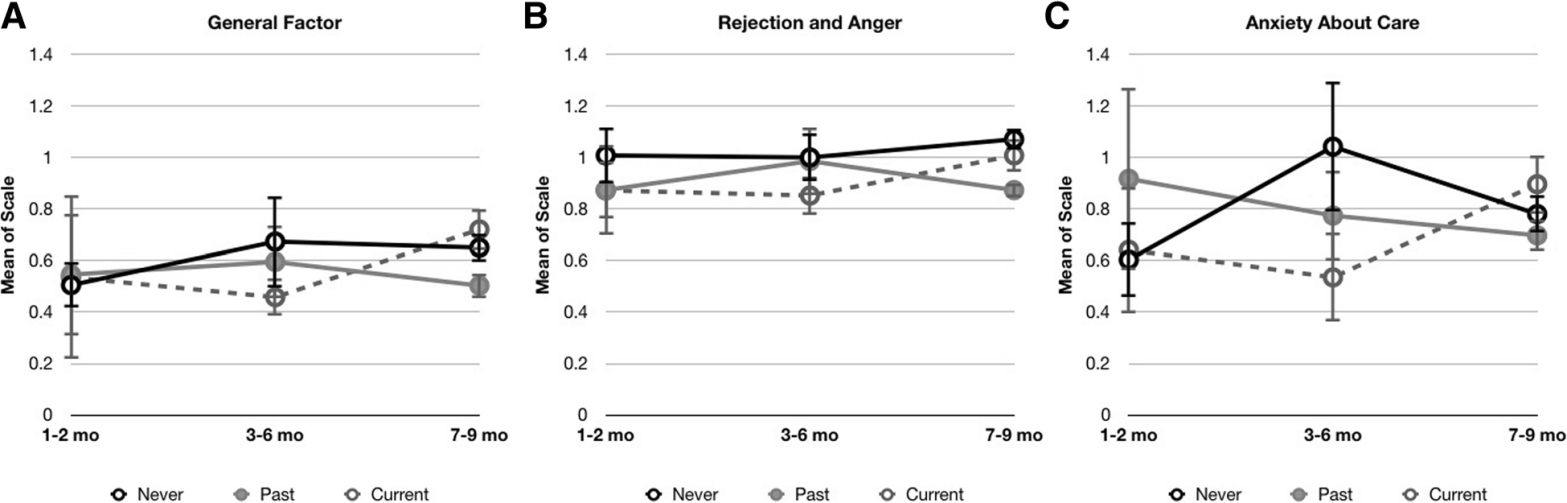
Results of ANOVAs on the subscales of the PBQ (**a**. General Factor; **b**. Rejection and Anger; **c**. Anxiety about care). The ordinate reflects the mean scores on each of the subscales, for each of the three breastfeeding groups (black line, ‘Never breastfed’ | full grey line and circle ‘Past breastfed’ | hashed gray line ‘currently breastfeeding, exclusive or partial’)

It remained possible that our second hypothesis – that the relationship between bonding and breastfeeding is age-dependent – would be supported. Hence, respondents were binned into three nearly equally-sized groups, according to infants’ ages (1–2 mo., *N* = 90; 3–6 mo., *N* = 91; 7–9 mo., *N* = 88). Due to the difference in size of breastfeeding groups, stratified bootstrap multivariate ANOVA was run with the three PBQ subscales, and a separate univariate ANOVA for the total PBQ scores; independent variables were infant age groups and breastfeeding groups. The multivariate ANOVA was not significant for any of the subscales (all *p*’s > 0.10), nor for the total PBQ score (*F* = _(2257)_ = 0.27, *p* = .784). There was no main effect of infant age group (Pillai’s Trace multivariate ANOVA: *F*_(6,512)_ = 0.18, *p* = .981; uni-ANOVA: *F*_(2,257)_ = 0.48, *p* = .617), nor for breastfeeding group (Pillai’s Trace *F*_(6,512)_ = .89, *p* = .502; uni-ANOVA: *F*_(2,257)_ = 0.03, *p* = .962), and there were no significant interactions (*F*’s < 1.0, see Fig. 1). Bayesian Factor ANOVAs were used to confirm the null results, yielding probabilities favoring the null hypothesis ranging from 1/BF10 from 0.14 to 0.356, i.e., between very strong to moderate odds favoring the null hypothesis ([44], Table 5).

**Table 5:** Breastfeeding variable with three levels (current | past | never); age group with three levels (1–2 mo | 3–6 mo | 7–9 mo)

Total PBQ	P(M)	P(M|data)	BF _M_	BF _10_	error %
Null model	0.250	0.732	8.210	1.000	
Breastfeeding group	0.250	0.049	0.153	0.066	0.024
Age group	0.250	0.204	0.767	0.278	0.024
Breastfeeding + Age	0.250	0.015	0.047	0.021	1.377
General Factor					
Null model	0.250	0.781	10.682	1.000	
Breastfeeding group	0.250	0.045	0.140	0.057	0.024
Age group	0.250	0.164	0.588	0.210	0.024
Breastfeeding + Age	0.250	0.011	0.033	0.014	3.003
Rejection & Anger					
Null model	0.250	0.751	9.071	1.000	
Breastfeeding group	0.250	0.146	0.514	0.195	0.027
Age group	0.250	0.084	0.276	0.112	0.023
Breastfeeding + Age	0.250	0.018	0.055	0.024	1.918
Anxiety About Care					
Null model	0.250	0.681	6.413	1.000	
Breastfeeding group	0.250	0.059	0.188	0.087	0.024
Age group	0.250	0.242	0.959	0.356	0.024
Breastfeeding + Age	0.250	0.017	0.053	0.025	1.034

*P(M)* prior model probabilities, *P(M|data)* the updated probabilities after the data, *BFM* Bayes Factor of model, reflects the degree to which the data have changed the prior odds, *BF10* odds for H1 (1/BF10 odds for null hypothesis). BF10 range 0.1–0.33 is considered moderate odds favoring the null hypothesis; 0.033–0.1 considered strong; below 0.033 is considered very strong odds [44]

As the PBQ is designed to assess bonding disorders, a separate analysis was run on a subscale created using the positive items only. The responses to these items were averaged and a univariate ANOVA was run with the three breastfeeding groups and three infant age groups as the independent factors. There were no main effects (breastfeeding groups *F*_(2,216)_ = 0.216, *p* = .806; infant age groups *F*_(2,216)_ = 0.377, *p* = .687), and no interaction (*F*_(2,216)_ = 1.896, *p* = .111, Additional file 1: Figure S2).

**Fig. 2:**
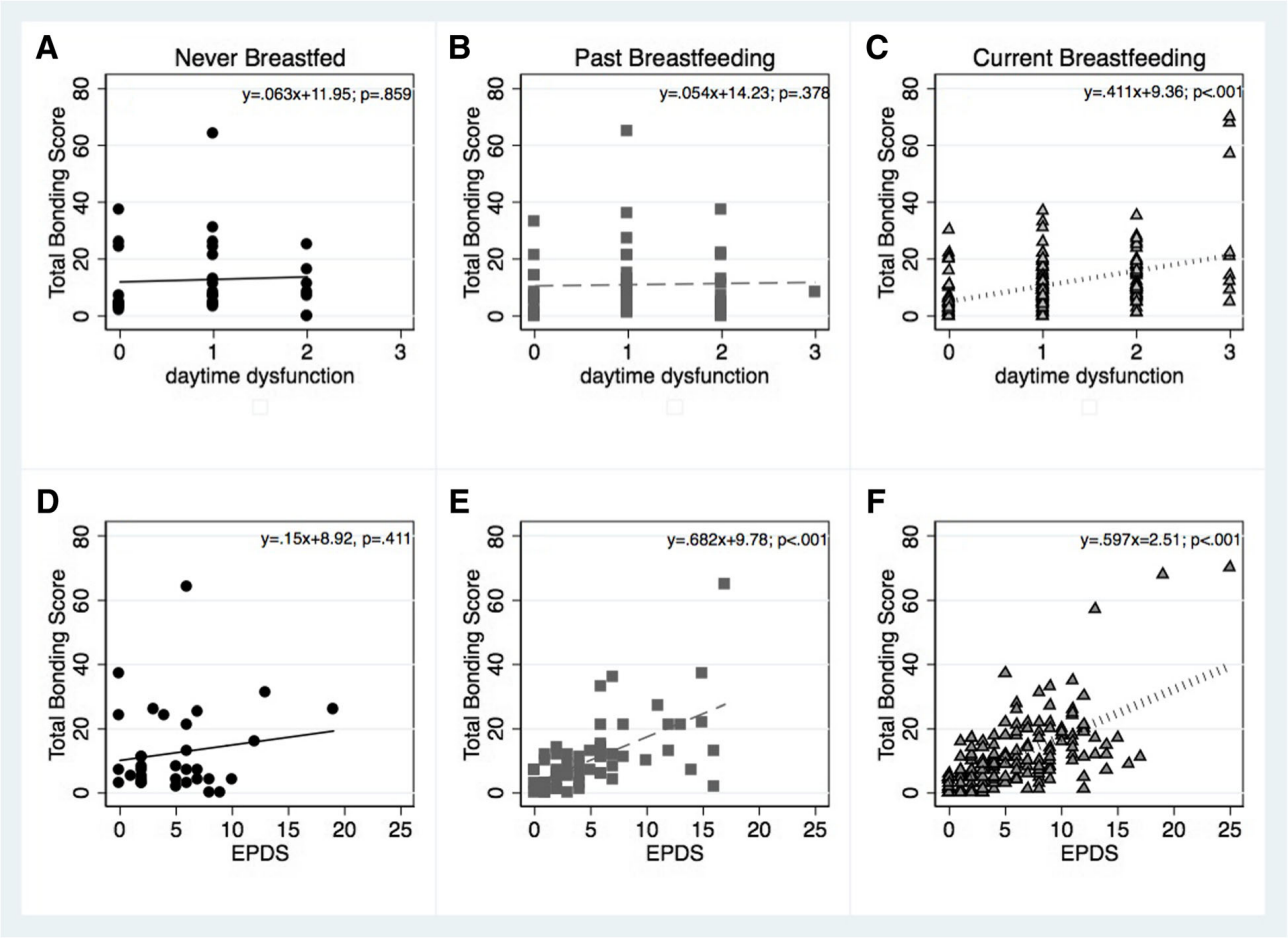
Scatter plots of the relationship between bonding (ordinate) and PSQI component of ‘daytime dysfunction’ (abscissa of **a**-**c**), and bonding with EPDS (abscissa of **d**-**f**), each plot representing a different category of breastfeeding (never, past, current). The formulas in each plot are of relationship between the factors

Our third hypothesis was that breastfeeding may moderate the deleterious effects of either sleep disturbance or depression symptoms on bonding. In this analysis, the daytime fatigue component of the PSQI (component 7) was used, as it was most strongly correlated with bonding. Infant age and duration of breastfeeding were used as background factors in the 1st level. “never breastfed” was the dummy factor contrasted with “past” (dummy 1) or with “current” (dummy 2). PSQI component 7 and EPDS - were centered, and their product with the breastfeeding group variable was used as the moderator in each of the models. As can be seen in Table 6, both models were significant, due to the positive correlation between bonding with sleep-related daytime symptoms (*F*_(6,263)_ = 4.91, *p* < .001) and with depressed mood (*F*_(6,263)_ = 19.27, *p* < .001). The effects of the daytime fatigue component on bonding was moderated by breastfeeding, such that for women who were currently breastfeeding there was a positive relationship between daytime fatigue and bonding, while for women who were not currently breastfeeding, or never nursed, bonding was unrelated to the daytime dysfunction component of the PSQI (Fig. 2a-c). Similarly, breastfeeding weakly moderated the relationship between depression and bonding, such that for women who never breastfed, the two measures were uncorrelated, but were correlated for past and current breastfeeding groups (Fig. 2d-f).

**Table 6:** Hierarchical regression analyses assessing the moderating effects of breastfeeding on the relationship between daytime fatigue (component #7 from the PSQI) with bonding (Top), and mood symptoms (EPDS) and bonding (Bottom)

	Level 1				Level 2				Level 3			
	B (SE)	Beta		CI95	B (SE)	Beta		CI95	B (SE)	Beta		CI95
Test of Moderation of Daytime Fatigue												
Baby age	.34 (.36)	.073		−.37, .11	.25 (.49)	.053		−.70, 1.21	.35 (.48)	.074		−.60, 1.31
Duration breastfed	−.12 (.35)	−.027		−.81, .57	−.10 (.54)	−.023		−1.17, .97	−.23 (.54)	−.051		−1.30, .83
PSQI component #7					3.8 (.84)	.276**		2.19, 5.49	−.78 (2.07)	−.056		−4.85, 3.30
BF dummy 1					−2.70 (2.42)	−.102		−7.45, 2.07	−1.51 (2.44)	−.057		−6.32, 3.31
BF dummy 2					−2.26 (2.88)	−.098		−7.92, 3.40	- .68 (2.92)	−.029		−6.43, 5.08
Moderator									2.92 (1.20)	.358*		7.16, 16.70
*R*^*2*^		.004				.081				.101		
*F R*^*2*^ change						7.36**				5.93*		
Test of Moderation of Depression Symptoms												
Baby age	.34 (.36)	.073		−.37, .11	.04 (.43)	.008		−.80,.88	.084 (.42)	.018		−.75, .92
Duration breastfed	−.12 (.35)	−.027		−.81, .57	−.23 (.48)	−.049		−1.16, .71	−.30 (.47)	−.065		−1.23, .64
EPDS					1.36 (.13)	.545**		1.10, 1.62	.79 (.32)	.318*		.17, 1.41
BF dummy 1					−1.72 (2.11)	−.065		−5.88, 2.44	−1.40 (2.11)	−.053		−5.56, 2.75
BF dummy 2					−1.26 (2.50)	−.005		−6.19, 3.66	−.76 (2.50)	−.033		−5.68, 4.17
Moderator									.37 (.19)	.249*		.001, .74
*R*^*2*^		.004				.295				.305		
*F R*^*2*^ change						36.38**				3.91*		

Each level reflects a step in the regression analysis. “never breastfed” was the dummy factor contrasted with “past” (dummy 1) and with “current” (dummy 2). * *p* < = .050; ** *p* < =.001

## Discussion

Contrary to our hypothesis, and to commonly held beliefs [6–11], breastfeeding was not associated with the quality of mother-infant bonding. Moreover, and in contrast with previous reports [26], breastfeeding did not attenuate the association between depression symptoms or sleep-related daytime symptoms with bonding. In fact, a positive association between mood symptoms and bonding difficulties was observed among mothers who were actively breastfeeding, but not among those who never breastfed or stopped breastfeeding. Although these findings warrant replication, they underscore the necessity for a better understanding of the emotional and social meanings of breastfeeding.

While the results regarding breastfeeding did not support our initial hypothesis, it should be noted that, in general, the outcome measures of this study conform to the current knowledgebase in the field. First, while the sample tended to be above the social-economical median of the general population, the percentage of breastfeeding mothers was commensurate with reported national averages [45]. Second, as in several previous studies, PBQ scores correlated both with the severity of depression symptoms and with sleep disturbance [24, 25, 46, 47]. Third, a positive relationship between income and sleep quality has been reliably demonstrated in several studies (e.g., [48]). With respect to the relationship of sleep and breastfeeding, as noted above, the few studies that assessed this relationship yielded mixed results. The observation that in this sample breastfeeding was associated with more sleep-related daytime fatigue, may be potentially due to factors not measured in the study, such as co-sleeping, partner support, and similar.

In the past several decades public health policies have actively promoted breastfeeding adducing three apparent evidence-based benefits to (1) the health and development of the infant (e.g., [49]), (2) the health of the mother [50, 51] and (3) the quality of the relationship between mother and infant (e.g., [12]). While medical and nutritional benefits of mother’s milk are well-established, direct evidence in support of a positive effect on maternal bonding is scant, at best [18]. It has been argued that implicit in the assumption that breastfeeding has positive effects on maternal bonding is the notion that lactation activates endocrine cues that reinforce engagement with the infant [18]. Oxytocin release, specifically, has received the most attention, being a key pro-social biological cue that enhances parental care in both human and non-human animals. However, recent evidence suggests that oxytocin is released by parents in response to many innate infant behaviors, such as clinging, facial expressions and vocal calls [52]. Feldman, Gordon, Influs, Gutbir & Ebstein (2013) [53] also showed stable oxytocin levels across a three-year period, concluding that: “*long-term stability of peripheral oxytocin suggests the notion that oxytocin represents a ‘trait-like’ dimension*”. Thus, breastfeeding-related oxytocin release may not have additive effects to oxytocin release associated with other infant-parent interactions.

Despite inconclusive empirical support, the bonding function of breastfeeding has permeated social meanings of motherhood [13, 54, 55] and is often cited as a major motivation for wanting to breastfeed (e.g., [7, 8]), as demonstrated in a recent meta-analysis of 17 ethnographic studies of women’s experiences and decision-making regarding breastfeeding, which included 500 women from six Western countries. The study found that the majority of women identified breastfeeding as “important for bonding”, that the belief that breastfeeding is consonant with being a “good mother” was highly prevalent, and that women who ceased to breastfeed experienced guilt and failure [56]. Thus, in Westernized cultures breastfeeding has become a “moral” choice [57], and a test of motherhood [15, 54, 55], while the psychological, social and economic costs to women have largely been ignored (e.g., [13, 17, 58]).

Our findings add to the handful of investigations that suggest that the link between breastfeeding and bonding is tenuous. It should be stressed that in this study bonding was measured using the PBQ, an instrument designed to assess bonding difficulties by gauging emotions and cognitions of mothers regarding their infant, including cognitions associated with parenting (e.g., “I feel trapped as a mother”), emotions towards the infant (e.g., “I love my baby to bits”), and anxiety (e.g., “my baby makes me feel anxious”). The PBQ is sensitive to maternal relational disturbances such as hostility, lack of emotion, and rejection of the infant which often coincide with depression and other psychopathology [25, 47, 59]. In this sense, the PBQ conforms with the definition of bonding as an affective state [3]. However, this raises the concern that the questionnaire stresses pathology over positive emotions. To address this concern, we further selectively analyzed only the positive items of the scale (α = 0.739), and this analysis also yielded no difference between the three breastfeeding groups. Thus, our findings suggest that in so far as the PBQ adequately measures mother-infant bonding, such bonding is likely achieved via multiple modes of interaction, in which the role of breastfeeding still needs to be established.

Other aspects of the mother-infant dyad may still be related to breastfeeding. For example, several studies found an association between breastfeeding and maternal responsivity [20, 60]. Tharner and colleagues [61] found that longer duration of breastfeeding was associated with greater maternal responsiveness, and more secure attachment in infants, although the authors noted that differences between breast- and bottle-fed groups were small, and maternal sensitivity did not correlate with infant attachment. Weaver and colleagues (2017) [62] reported an association between breastfeeding duration and maternal sensitivity, in a longitudinal study spanning 10 years. Similarly, in a fMRI study, breastfeeding mothers had stronger activation in regions implicated in caregiving and empathy when listening to their own infant cry, compared to formula-feeding mothers; suggesting that breastfeeding facilitates attunement to infant signaling. It should be stressed, however, that bonding measures were not investigated in these studies, and studies to identify the association of bonding with measures of either responsivity or infant attachment are lacking.

Several limitations of this study should be mentioned. First, failing to reject the null hypothesis does not typically allow to conclude that the null hypothesis is supported. However, given that other findings match current literature, that others [20, 22] have reported similar null effects, and the confirmation of the results using Bayesian statistics, we are confident that these results are not a false negative. Second, as the study was a cross-sectional assessment, the effects of breastfeeding on individual mothers, over time, is unknown, and merit further investigation. Third, while the categories used in the study conform with recommended definitions (e.g., [63]), no differentiation was made between actual breastfeeding and feeding with expelled mother’s milk. Arguably in the context bonding, feeding expelled mother’s milk may be behaviorally more similar to bottle-feeding. More accurate categories for breastfeeding would help clarify this issue. Fourth, it should be further noted that although the sample was representative of the distribution of infant feeding strategies [45], and large enough to allow for the analytical methods employed, the number of non-breastfeeding mothers was small. Further, being a convenience sample, and an internet-based protocol, the distribution of socio-economic characteristics was not representative of the larger population. Finally, mother-infant bonding is a complex set of emotions and cognitions, and the instrument used in this study to assess bonding was originally designed for detecting bonding disorders, future studies may prefer instruments that focus on positive aspects of the maternal emotions towards her infant. While we attempted to overcome this limitation by selectively analyzing only the positive items in the PBQ, such a scale has not been independently validated in the literature. Future studies should employ longitudinal measurements, on larger, more representative, samples, assessing both breastfeeding as well as bonding and other measurements of mother-infant relationship.

## Conclusions

In this study, breastfeeding was not associated with the quality of mother-infant bonding, nor did it attenuate the association between mood and sleep difficulty symptoms with bonding. These observations indicate that while breastfeeding may be beneficial to infant’s and mother’s health, caution should be used when arguing that it promotes the maternal bond. Arguably, the information provided to parents regarding the benefits of breastfeeding as to the nutritional and medical values of breastfeeding as well as to the emotional values should be accurate and evidence-based, and mothers may be reassured that we don’t currently have evidence that their bond with their child will be negatively impacted if they do not breastfeed. This may help reduce further stigmatization and guilt regarding the bonding process among mothers who choose not to or are unable to breastfeed. Nevertheless, as studies regarding breastfeeding and bonding and other measurements of parent-infant relationship are scarce, further research is clearly needed before definite conclusions can be drawn.

## Additional file

Additional file 1: **Figure S1.** distribution of breastfeeding groups across the different ages in the sample, demonstrating a decrease over time in the propostion of women who report exclusive breastfeeding, and an increase of partial and past feeding. **Figure S2.** results of an ANOVA comparing the effects of breastfeeding category and infant age on the average responses to the positive items only of the Postpartum Bonding Questionnaire (PBQ). (DOCX 974 kb)

## Abbreviations

BF: Bayes Factor
EPDS: Edinburgh Postnatal Depression Scale
PBQ: Postpartum Bonding Questionnaire
PSQI: Pittsburgh Sleep Quality Index
SOL: Sleep Onset Latency
TST: Total Sleep Time
WASO: Wake After Sleep Onset
