# Supplementary material for: Mother-infant bonding is not associated with feeding type: a community study sample
Source: BMC Pregnancy Childbirth. 2019 Apr 11;19:125. doi: 10.1186/s12884-019-2264-0 (PMC6458683; doi:10.1186/s12884-019-2264-0)
Supplement: Supplementary file 1 — Figure S1. distribution of breastfeeding groups across the different ages in the sample, demonstrating a decrease over time in the propostion of women who report exclusive breastfeeding, and an increase of partial and past feeding. Figure S2. results of an ANOVA comparing the effects of breastfeeding category and infant age on the average responses to the positive items only of the Postpartum Bonding Questionnaire (PBQ). (DOCX 974 kb) [file 12884_2019_2264_MOESM1_ESM.docx]

**Supplemental Figure 1**

Distribution of the breastfeeding categories as a function of infant age, represented in percentage of the sample.

**Supplemental Figure 1**


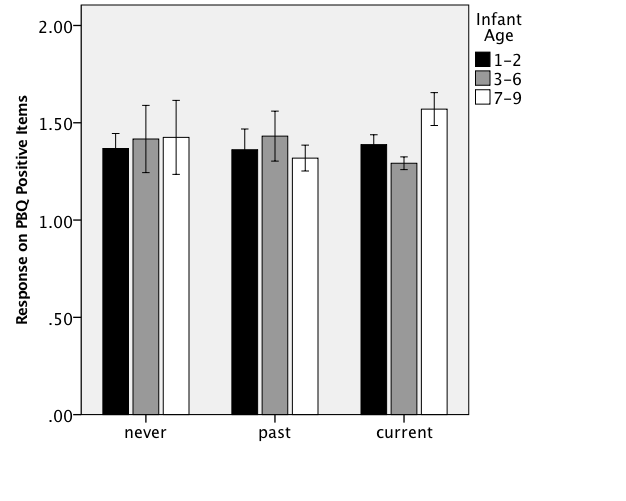


Means and standard errors of the average responses to the eight positive items on the PBQ. The Groups depict the three breastfeeding categories (exclusive or partial, N=178), past (N=60), and never nursed (N=33), according to the infant age groups in months.

|  | *df* | *F* | *p* |
| --- | --- | --- | --- |
| Corrected Model | 8 | 1.655 | .110 |
| Intercept | 1 | 1513.072 | .000 |
| Breastfeeding group | 2 | 0.216 | .806 |
| Age group | 2 | 0.377 | .687 |
| Interaction | 4 | 1.896 | .111 |
| Error | 262 |  |  |
| Total | 271 |  |  |
| Corrected Total | 270 |  |  |
